# Constructing an S-Scheme NiO/SrTiO_3_ Heterojunction for Highly Enhanced Visible-Light Photocatalytic Removal of Methylene Blue

**DOI:** 10.3390/ma19050845

**Published:** 2026-02-25

**Authors:** Hongfei Wu, Yanlong Gao, Senwei Wu, Xiujian Zhao, Yi Xie, Shouqin Tian

**Affiliations:** 1 State Key Laboratory of Advanced Glass Materials, Wuhan University of Technology, Wuhan 430070, China; 13753131311@163.com (H.W.); 15164661794@163.com (Y.G.); senwei_wu@whut.edu.cn (S.W.); 2 State Key Laboratory of Silicate Materials for Architectures, Wuhan University of Technology, Wuhan 430070, China; opluse@whut.edu.cn (X.Z.); xiey@whut.edu.cn (Y.X.)

**Keywords:** strontium titanate, nickel oxide, heterojunction, photocatalysis, methylene blue degradation

## Abstract

Organic dye pollution in industrial wastewater poses a serious environmental challenge, with methylene blue (MB) serving as a typical persistent pollutant due to its stable chemical structure, recalcitrance to degradation, and eco-toxicity. Conventional physical, chemical, and biological treatment methods suffer from limitations such as insufficient efficiency, high cost, or the tendency to generate secondary pollution. Based on green and sustainable photocatalysis technology, this study designed and prepared a NiO/SrTiO_3_ p-n heterojunction photocatalysts, aiming to broaden the light-response range and enhance charge-carrier separation efficiency. The optimal sample (NiO (10%)/SrTiO_3_) achieved complete photocatalytic degradation of MB within 9 min, with an apparent rate constant 34.6 times that of pure SrTiO_3_. It also showed good cyclic stability. Trapping experiments confirmed that •OH and •O_2_^−^ were the key active species in the degradation process. Combined with band structure and PL analyses, an S-scheme charge-transfer mechanism was proposed, clarifying the critical role of the built-in electric field at the heterojunction interface in promoting carrier separation while maintaining high redox capability. This work not only provides a new pathway for developing efficient and stable SrTiO_3_-based photocatalysts but also offers theoretical and experimental support for the practical application of p-n heterojunction photocatalysts in environmental pollution control.

## 1. Introduction

Industrial development, while promoting social progress, has also intensified environmental pollution. Among various issues, water contamination caused by organic dyes, pesticides, and other chemicals is particularly severe and has become a global challenge [1,2]. As a fundamental industry, the textile sector has emerged as one of the major sources of water pollution due to its large-scale production [3]. The wastewater discharged contains a significant amount of refractory organic compounds, especially synthetic dyes, posing a persistent threat to aquatic ecosystems [4]. Among these, the cationic dye Methylene Blue (MB) is widely used owing to its vivid color and strong fixation properties, leading to its frequent presence in various water bodies [5]. MB belongs to the phenothiazine class of dyes, characterized by a stable tricyclic conjugated π-structure and hydrophilic dimethylamino groups. These features grant it both good water solubility and chemical stability, classifying it as a typical “persistent” pollutant [6,7]. Its environmental hazards are multifaceted: ecologically, even low concentrations of MB can increase water color, hinder light transmission, inhibit photosynthesis, and disrupt dissolved oxygen levels and primary productivity in aquatic systems [8,9]; in terms of health risks, MB can cause irritation through skin contact, and its metabolites may interfere with cellular redox processes, leading to potential in vivo toxicity [10,11]. Therefore, developing efficient, economical, and environmentally friendly technologies for MB removal holds significant importance for water environment remediation and public health protection.

Currently, the treatment of MB-containing wastewater primarily relies on physical, chemical, and biological methods. Physical approaches such as adsorption [12,13], precipitation [14], and membrane separation [15,16] remove pollutants through phase separation. While these methods are relatively simple and low-cost, they face challenges including difficult adsorbent regeneration, membrane fouling, and the need for secondary treatment of concentrated waste streams, failing to achieve complete degradation of pollutants. Chemical methods like advanced oxidation [17,18], ozonation [19], and electrochemical processes [20] can break down dye molecules via strong oxidation. However, they often suffer from limitations such as high reagent consumption, elevated costs, the potential generation of toxic by-products, or electrode passivation. Biological methods utilize microorganisms or plants to metabolize and degrade pollutants [21,22,23]. Although environmentally friendly and energy-efficient, they generally involve long treatment cycles, are sensitive to water quality conditions, and often exhibit limited degradation efficiency for dyes like MB, which exhibits strong biotoxicity. In summary, conventional methods present shortcomings in terms of efficiency, cost, or environmental compatibility.

In contrast, semiconductor photocatalysis technology, which utilizes solar energy as the driving force, can generate reactive species to degrade organic pollutants under mild conditions without causing secondary pollution. It is thus regarded as a green and sustainable approach for pollution remediation [24]. The core of this technology lies in the design and preparation of high-performance photocatalytic materials. Among the various photocatalysts, the perovskite oxide strontium titanate (SrTiO_3_) has attracted considerable attention due to its excellent chemical stability, tunable band structure, low environmental toxicity, and cost-effectiveness [25]. The positions of its conduction and valence bands endow it with the potential to drive both oxidation and reduction reactions, making it suitable for pollutant degradation. However, pristine SrTiO_3_ possesses a relatively wide band gap (approximately 3.2 eV), enabling it to harness only about 5% of the ultraviolet region in the solar spectrum. Furthermore, its rapid recombination rate of photogenerated charge carriers severely limits its practical application efficiency [26].

To overcome these limitations, constructing heterojunctions represents one of the effective strategies. By coupling SrTiO_3_ with another semiconductor, its light absorption range can be extended into the visible region. Moreover, the built-in electric field at the interface can promote the separation and migration of photogenerated electron-hole pairs, thereby significantly enhancing the photocatalytic performance [27]. For instance, the MoS_2_/SrTiO_3_ Z-scheme heterojunction constructed by Rezaei et al. [28] exhibited an 8-fold higher degradation efficiency for Rhodamine B compared to pure SrTiO_3_; the SrTiO_3_/BiOBr heterojunction reported by Wang et al. [29] achieved a 94.8% degradation rate for oxytetracycline under visible light. These studies confirm the effectiveness of heterojunction engineering in improving the photocatalytic activity of SrTiO_3_. Among various candidate semiconductors, nickel oxide (NiO), a p-type semiconductor, has attracted widespread interest in photoelectrics and catalysis due to its suitable band gap, high hole mobility, and good photochemical stability [30,31]. Combining p-type NiO with n-type SrTiO_3_ to form a p-n heterojunction is expected to create a stronger built-in electric field, further accelerating charge separation. Furthermore, appropriate band alignment may facilitate the formation of S-scheme or Z-scheme charge transfer pathways, which can inhibit recombination while maintaining high redox capabilities, ultimately enabling efficient pollutant degradation [32].

This study reports the rational design and fabrication of NiO/SrTiO_3_ p-n heterojunction photocatalysts for MB degradation. In contrast to conventional mechanical mixing or coprecipitation methods, a stepwise hydrothermal-controlled coupling strategy was adopted to construct a tightly bonded NiO/SrTiO_3_ heterointerface. We systematically investigate the structure, interfacial properties, and the degradation performance of this heterojunction towards MB under visible light and further elucidate the underlying photocatalytic reaction mechanism. This research not only provides a novel strategy for developing highly efficient and stable SrTiO_3_-based photocatalysts but also offers experimental evidence for constructing high-performance p-n heterojunction systems, demonstrating potential application value in the field of environmental pollution control.

## 2. Experiment and Characterization

### 2.1. Reagents

All chemicals used in this study were of analytical grade. Tetrabutyl titanate (C_16_H_36_O_4_Ti, TBOT, 98%), ethylene glycol (C_2_H_6_O_2_, EG), sodium hydroxide (NaOH), absolute ethanol (C_2_H_5_OH), oxalic acid dihydrate (H_2_C_2_O_4_·2H_2_O), isopropanol (IPA), and benzoquinone (BQ) were purchased from China National Pharmaceutical Group Chemical Reagent (Shanghai, China) Co., Ltd. Strontium nitrate (Sr(NO_3_)_2_) and nickel nitrate hexahydrate (Ni(NO_3_)_2_·6H_2_O) were obtained from Aladdin Reagent (Shanghai, China) Co., Ltd. Deionized water was produced in the laboratory.

### 2.2. Synthesis of NiO/SrTiO_3_ Photocatalysts

#### 2.2.1. Preparation of SrTiO_3_

A total of 2.5 mmol of tetrabutyl orthotitanate (TBOT) was added to 60 mL of ethylene glycol (EG) under stirring. Meanwhile, 2.5 mmol of Sr(NO_3_)_2_ was dissolved in 5 mL of deionized water with thorough mixing. The Sr(NO_3_)_2_ aqueous solution was then slowly dripped into the TBOT/EG mixture under continuous stirring, followed by the addition of 2.5 mL of a 10 M NaOH solution. The resulting homogeneous mixture was transferred into a Teflon-lined autoclave and reacted under hydrothermal conditions at 180 °C for 24 h. Finally, the product was collected and washed repeatedly with deionized water and ethanol via centrifugation, and then dried at 80 °C to obtain SrTiO_3_ powder.

#### 2.2.2. Preparation of NiO/SrTiO_3_

An appropriate amount of nickel nitrate hexahydrate (Ni(NO_3_)_2_·6H_2_O) was dissolved in 20 mL of deionized water, followed by the addition of 30 mL of ethylene glycol (EG) under continuous stirring. A suitable quantity of H_2_C_2_O_4_·2H_2_O was then introduced. After thorough stirring, a predetermined amount of the as-synthesized SrTiO_3_ powder was added to the solution. The homogeneous mixture was transferred into a Teflon-lined autoclave and reacted under hydrothermal conditions at 120 °C for 12 h. The resulting product was collected, repeatedly washed via centrifugation with deionized water and ethanol, and dried at 80 °C. The obtained powder was placed in a crucible and annealed at 450 °C for 2 h in air with a heating rate of 5 °C·min^−1^, yielding the final NiO/SrTiO_3_ catalysts.

### 2.3. Characterization

X-ray diffraction (XRD) patterns of the samples were obtained from a Bruker D8 Advance diffractometer (Karlsruhe, Germany) with Cu Kα radiation at a rated power of 3 kW. Data were collected over a 2θ range of 10–80° with a scanning speed of 10° min^−1^. The morphology of the samples was characterized by field emission scanning electron microscopy (FESEM) on a Zeiss Ultra Plus microscope (Carl Zeiss AG, Oberkochen, Germany). The microstructure and elemental distribution were further analyzed using field-emission high-resolution transmission electron microscopy (HRTEM, JEM-2100F, JEOL, Tokyo, Japan) equipped with an energy-dispersive X-ray spectroscopy (EDS) system. X-ray photoelectron spectroscopy (XPS) measurements were performed on a Thermo Scientific K-Alpha spectrometer (FL, USA). Ultraviolet-visible (UV-Vis) diffuse reflectance spectra (DRS) were collected on a PerkinElmer Lambda 750 S spectrophotometer (CT, USA). Photoluminescence (PL) spectra were obtained on a time-resolved fluorescence spectrometer (QM/TM/NIR).

### 2.4. Photocatalytic Experiments

A total of 50 mg of the photocatalyst was dispersed in a quartz glass reactor containing 50 mL of an aqueous methylene blue (MB) solution (initial concentration: 10 mg L^−1^, pH = 7). A 500 W xenon lamp equipped with a 420 nm cut-off filter was used as the simulated solar light source. The mixture was first stirred in the dark for 30 min to establish an adsorption–desorption equilibrium. During the illumination period, approximately 3 mL of the suspension was sampled at 3 min intervals and immediately filtered through a syringe filter with a 0.22 μm membrane to remove the catalyst particles. The degradation of methylene blue was evaluated by monitoring the decrease of its characteristic absorption peak at 667 nm using a UV-Vis spectrophotometer (UV-2600, Shimadzu, Kyoto, Japan).

## 3. Results and Discussion

### 3.1. Structural Characterization of NiO/SrTiO_3_ Series Samples

**Figure 1:**
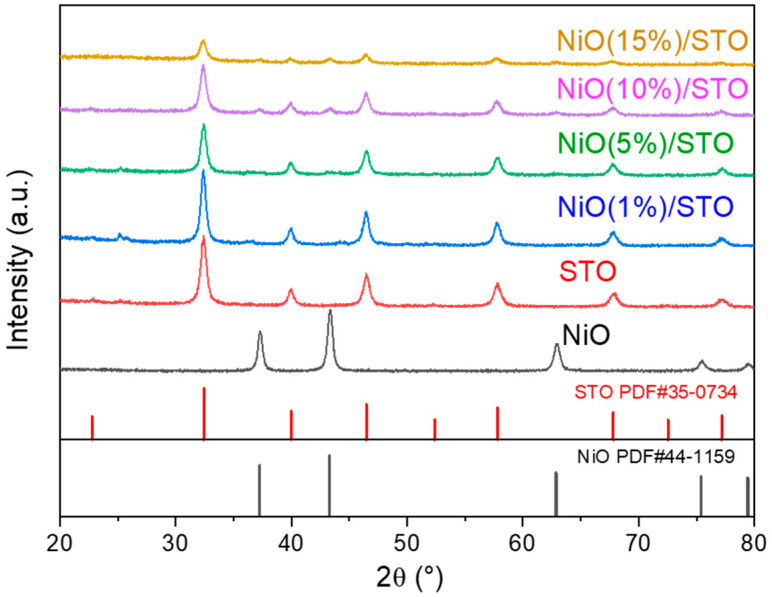
XRD patterns of the NiO/SrTiO_3_ samples.

Figure 1 shows the XRD patterns of the NiO/SrTiO_3_ samples. All samples exhibit distinct diffraction peaks at 2θ of 32.35°, 39.91°, 46.42°, 57.72°, 67.74°, and 77.09°, which correspond to the (110), (111), (200), (211), (220), and (310) crystal planes of the cubic SrTiO_3_ structure (JCPDS card no. 05-0734) [33], confirming the successful synthesis of pure-phase cubic SrTiO_3_. Furthermore, the pure NiO sample shows five prominent diffraction peaks at 2θ of 37.25°, 43.29°, 62.85°, 75.40°, and 79.37°, corresponding to the (101), (012), (110), (113), and (202) planes of cubic-phase NiO (JCPDS#44-1159) [32]. In the XRD patterns of the NiO/SrTiO_3_ samples, diffraction peaks at 37.25°, 43.29°, and 62.85° are observed, which correspond to the (101), (012), and (110) crystal planes of NiO, respectively. This indicates the coexistence of NiO and SrTiO_3_ in the sample. With increasing NiO content, the characteristic diffraction peaks of SrTiO_3_ show no obvious shift, while their intensities gradually decrease, which can be attributed to surface coverage by NiO and compositional effects rather than lattice distortion. These XRD features demonstrate that the introduction of NiO mainly affects the interfacial structure instead of the bulk crystal structure of SrTiO_3_. Furthermore, the weak diffraction peaks observed at approximately 25° and 36° can be attributed to trace SrCO_3_ ((JCPDS#05-0418), likely formed due to surface reaction of residual ethylene glycol with Sr species during annealing. The impurity phase is present in a negligible amount and does not affect the primary crystal structure.

**Figure 2:**
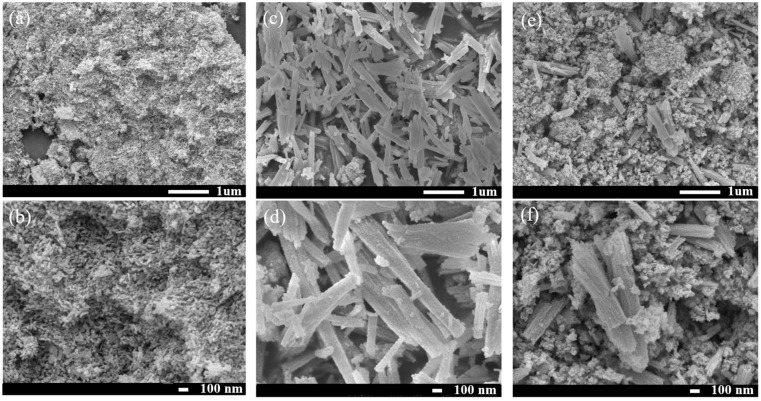
SEM images of the samples: (**a**,**b**) SrTiO_3_; (**c**,**d**) NiO; (**e**,**f**) NiO (10%)/SrTiO_3_ (hereinafter abbreviated as NiO (10%)/STO).

SEM results (Figure 2) clearly reveal the surface morphology and microstructure of the different samples. The SrTiO_3_ sample (Figure 2a,b) exhibits an irregular sheet-like morphology formed by the agglomeration of short rod-shaped particles. The NiO sample (Figure 2c,d) displays a typical plate-like structure with thin layers and distinct edges. At the micrometer scale (~1 μm), the NiO predominantly exhibits a plate-like morphology with a size of approximately 700 nm. Upon higher-magnification observation (~100 nm), the surfaces of the NiO plates display a distinct rough texture with irregular protrusions, resembling a bitter-gourd-like morphology. The short rod-like features observed in the SEM images are attributed to incompletely grown NiO rather than to the presence of a separate crystalline phase. Moreover, a statistical particle size analysis (sample size > 200) was carried out for both SrTiO_3_ and NiO. The resulting histogram (Figure S1) indicates that SrTiO_3_ particles have an average size of ~60 nm, while NiO mainly forms plate-like structures with characteristic dimensions of approximately 700 nm. In the NiO (10%)/SrTiO_3_ sample (Figure 2e,f), the coexistence and integration of both characteristic morphologies can be observed: the agglomerated short rod-shaped SrTiO_3_ and the plate-like NiO are interwoven and in close contact.

**Figure 3:**
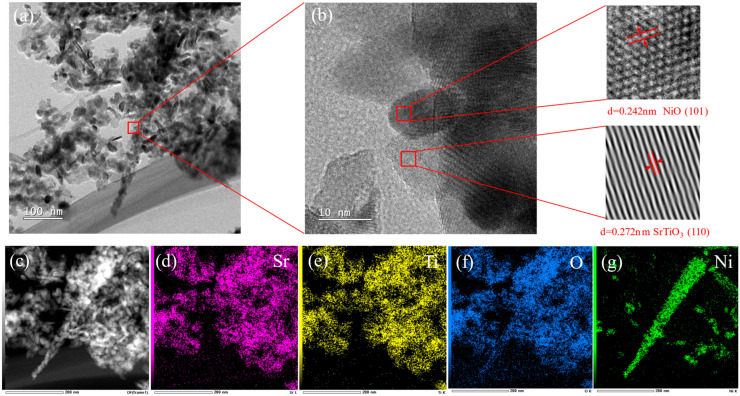
(**a**) TEM image, (**b**) HRTEM image, and (**c**–**g**) EDS elemental mapping of NiO (10%)/STO sample: (**d**) Sr, (**e**) Ti, (**f**) O, (**g**) Ni.

Figure 3a presents the TEM image of NiO (10%)/STO sample, in which the composite morphology of short rod-like SrTiO_3_ agglomerates and sheet-like NiO can be clearly distinguished. The SrTiO_3_ particles exhibit a relatively small size, suggesting a potentially high specific surface area and abundant surface active sites. NiO shows a typical two-dimensional sheet-like structure, interwoven and in intimate contact with SrTiO_3_. Moreover, the NiO plates exhibit a rough surface texture characterized by irregular nanoscale protrusions, resembling a bitter-gourd-like morphology. The HRTEM image (Figure 3b) further reveals the lattice structure at the interface. Clear lattice fringes with spacings of 0.272 nm and 0.242 nm are observable, corresponding to the (110) plane of cubic SrTiO_3_ [34] and the (101) plane of cubic NiO [35], respectively. This result provides direct evidence for the successful construction of the NiO/SrTiO_3_ heterojunction. The EDS elemental mapping images (Figure 3d–g) show the distribution of four elements (Sr, Ti, O, and Ni) within the NiO (10%)/STO sample. The signals for Sr, Ti, and O are uniformly distributed across the observed region, and their spatial coverage closely matches the morphology of the short rod-like agglomerates seen in the TEM image, corresponding to SrTiO_3_. In contrast, the distribution of Ni exhibits a distinct sheet-like pattern, consistent with the plate-like NiO morphology observed in the TEM results. This directly confirms the successful loading of NiO onto the SrTiO_3_ surface and their intimate spatial combination at the nanoscale, further supporting the construction of the NiO/SrTiO_3_ heterojunction structure. Such an intimate interfacial contact is crucial for promoting charge separation, which directly contributes to the enhanced photocatalytic activity.

**Figure 4:**
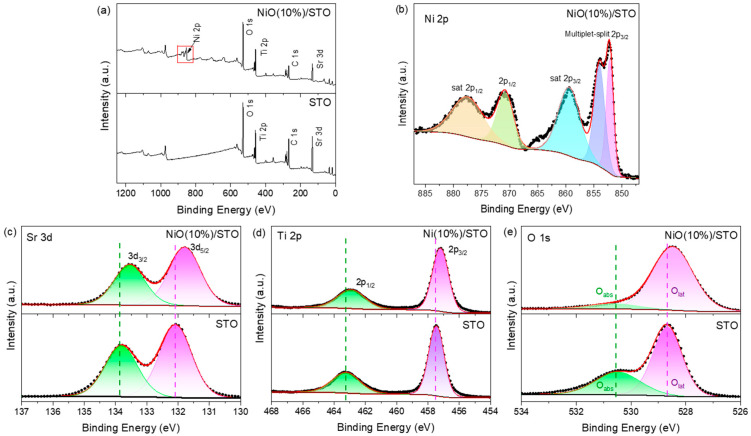
(**a**) XPS survey spectra of SrTiO_3_ and the NiO (10%)/STO sample. (**b**) High-resolution Ni 2p spectrum of the NiO (10%)/STO sample. (**c**–**e**) High-resolution Sr 3d, Ti 2p, and O 1s spectra of pure SrTiO_3_ and the NiO (10%)/STO sample, respectively.

XPS analysis was conducted to investigate the surface chemical states of the samples. All spectra were charge-corrected by C 1s peak at 284.80 eV. The XPS survey spectra (Figure 4a) confirm the presence of C, Sr, Ti, and O elements in both the pure SrTiO_3_ and NiO (10 wt%)/SrTiO_3_ samples. The NiO (10 wt%)/SrTiO_3_ sample exhibits a distinct Ni 2p characteristic peak at approximately 854 eV, indicating the introduction of NiO. The high-resolution Ni 2p spectrum is displayed in Figure 4b. The Ni 2p_3/2_ region shows two peaks at 852.5 eV and 854.1 eV, which correspond to multiplet-split of Ni^2+^ 2p_3/2_, along with a satellite peak at 859.6 eV. The peaks at 872.2 eV and 878.5 eV are attributed to Ni^2+^ 2p_1/2_ and its associated satellite peak, respectively [36], indicating that nickel predominantly exists in the form of NiO. Figure 4c shows the Sr 3d XPS peaks at 132.1 eV and 139.8 eV, corresponding to the Sr 3d_5/2_ and Sr 3d_3/2_ electronic states of Sr^2+^ [37]. In the high-resolution Ti 2p spectrum (Figure 4d), the double peaks at 463.3 eV and 457.4 eV can be assigned to the Ti 2p_1/2_ and Ti 2p_3/2_ states of Ti^4+^ [38,39]. Notably, compared to pure SrTiO_3_, the NiO (10 wt%)/SrTiO_3_ sample shows discernible shifts in the binding energies of both the Sr 3d and Ti 2p peaks, suggesting electronic interaction between NiO and SrTiO_3_. In the O 1s spectrum (Figure 4e), the peaks at 528.8 eV and 530.6 eV are attributed to lattice oxygen (O_lat_) and adsorbed oxygen species (O_ads_), respectively [40]. The shift in the lattice oxygen peak of the NiO (10 wt%)/SrTiO_3_ sample compared to pure SrTiO_3_ further indicates an alteration in the local electronic environment around the oxygen atoms resulting by the introduction of NiO [41]. The binding energy shifts observed in the Sr 3d, Ti 2p, and O 1s spectra indicate strong interfacial electronic coupling between NiO and SrTiO_3_, providing chemical-state evidence for Fermi level equilibration and the establishment of a built-in electric field at the heterointerface. Collectively, the XPS results confirm the successful construction of the NiO/SrTiO_3_ heterojunction and substantiate the presence of pronounced interfacial charge redistribution. Such electronic coupling is crucial for facilitating efficient charge separation and suppressing carrier recombination, thereby accounting for the significantly enhanced photocatalytic performance.

### 3.2. Optical and Photocatalytic Properties

**Figure 5:**
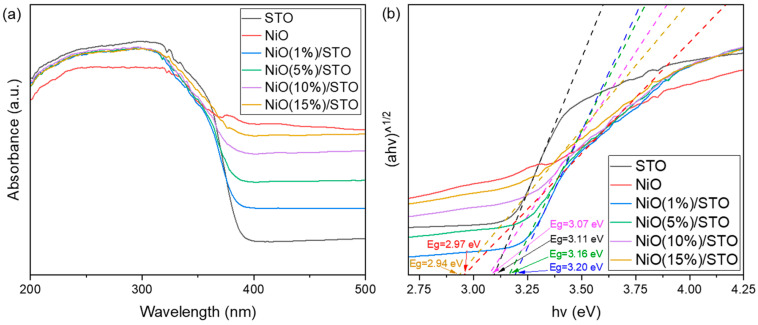
(**a**) UV-Vis absorption spectra and, (**b**) the derived optical band gaps of the NiO/SrTiO_3_ samples.

The optical absorption properties of pure SrTiO_3_, NiO, and NiO/SrTiO_3_ samples were investigated using DRS. As shown in Figure 5a, pure SrTiO_3_ exhibits absorption only below 400 nm, indicating its responsiveness solely to ultraviolet light. In contrast, the NiO/SrTiO_3_ samples display enhanced absorption intensity in the visible-light region (>400 nm), demonstrating that the formation of the heterojunction effectively extends the light-response range of the material [42]. The bandgap energies (E_g_) of the samples were estimated by extrapolating the linear portion of plots based on the Kubelka-Munk function versus photon energy [43]. The optical bandgap of pure SrTiO_3_ is 3.11 eV, close to its theoretical value (3.2 eV), while that of NiO is 2.97 eV. With increasing NiO loading, the bandgap of the composite materials shows a gradual narrowing trend. This variation indicates that the introduction of NiO modifies the band structure of SrTiO_3_, which contributes to improved visible-light harvesting capacity and thereby provides an optical foundation for enhanced photocatalytic activity [35].

**Figure 6:**
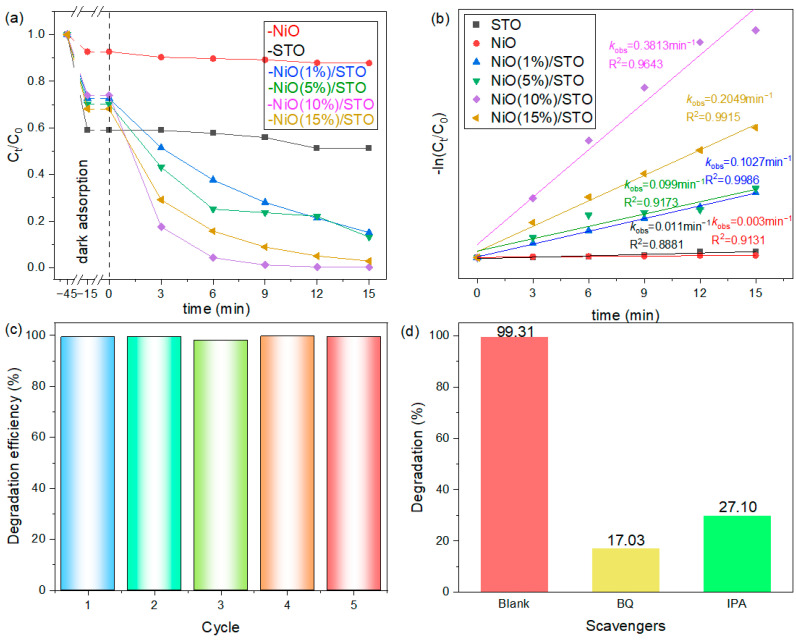
(**a**) Adsorption and photodegradation capabilities of the samples for MB. (**b**) Plots of −ln(C_t_/C_0_) versus irradiation time (*t*) for the samples. (**c**) Cycling performance of the NiO (10%)/STO sample. (**d**) Trapping experiments using different scavengers for the NiO (10%)/STO catalyst.

Figure 6a compares the photocatalytic degradation performance of different samples towards methylene blue (MB). Prior to irradiation, the suspension was stirred in the dark to establish adsorption–desorption equilibrium, thereby eliminating the influence of physical adsorption to the photocatalytic degradation. After 15 min of irradiation, almost no degradation activity for MB was observed with either pure SrTiO_3_ or pure NiO samples. In contrast, the photocatalytic performance of the NiO/SrTiO_3_ samples initially increased and then decreased with the increasing NiO loading. Among them, NiO (1%)/STO and NiO (5%)/STO degraded approximately 67% of MB within 15 min. The optimal sample, NiO (10%)/STO, achieved complete degradation of MB in just 9 min. When the NiO loading was further increased to 15%, the degradation rate decreased, requiring 15 min for complete degradation of MB. The corresponding apparent photocatalytic rate constants (k) were calculated using the pseudo-first-order kinetics model [44]: −ln(C_t_/C_0_) = kt, where C_t_ and C_0_ represent the MB concentration at time t and the initial concentration, respectively. As shown in Figure 6b, the NiO (10%)/STO sample exhibited the highest k value (0.3813 min^−1^), which is approximately 34.6 times that of the pure SrTiO_3_ sample (k = 0.011 min^−1^), highlighting the significant catalytic enhancement effect of the heterojunction structure. Cycling results (Figure 6c) further confirmed the high stability of the catalyst, as no significant activity loss was observed after five consecutive runs, indicating its promising application potential in photocatalytic wastewater treatment. Since hydroxyl radicals (•OH) and superoxide anion radicals (•O_2_^−^) are the primary reactive species involved in the photocatalytic degradation of pollutants [45], isopropanol (IPA) and benzoquinone (BQ) were introduced into the reaction system as scavengers for •OH and •O_2_^−^, respectively, to identify the dominant active species [46]. As shown in Figure 6d, the degradation efficiency of MB reached 99.31% in the absence of any scavenger. When IPA and BQ were added, the degradation efficiency decreased to 27.10% and 17.03%, respectively, indicating that both •OH and •O_2_^−^ play active roles in the process [47]. (Detailed experimental procedures for the reactive species trapping experiments are available in the Supporting Information). Furthermore, the prepared NiO (10 wt%)/SrTiO_3_ sample exhibits superior catalytic activity for organic pollutant degradation compared with other reported SrTiO_3_-based heterojunction photocatalysts in Table 1. (Due to variations in experimental conditions among different studies, the data listed below are intended for qualitative comparison of performance trends.)

### 3.3. Photocatalytic Mechanism

**Figure 7:**
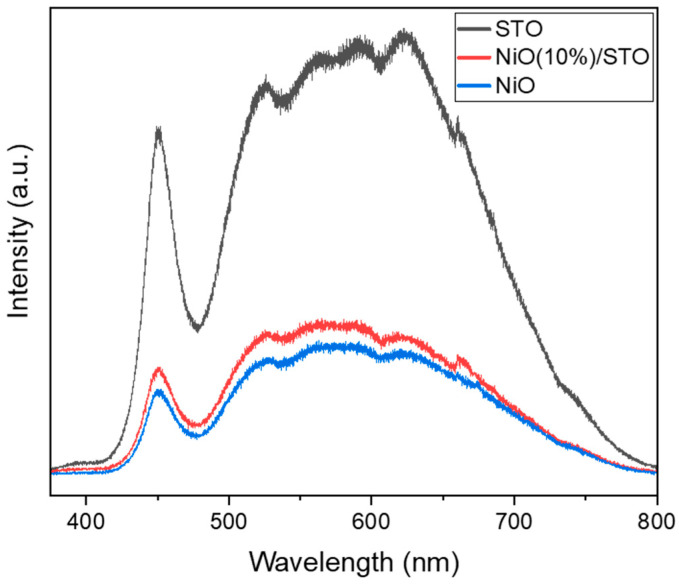
PL spectra of SrTiO_3_, NiO, and NiO (10%)/STO.

The PL spectra of SrTiO_3_, NiO, and NiO (10%)/STO were collected to investigate the behavior of charge carriers (Figure 7). The strong emission peak around 450 nm is attributed to band-to-band recombination, consistent with the intrinsic emission of SrTiO_3_ [55]. NiO exhibits low luminescence intensity, aligning with previous reports [41]. Notably, the PL intensity of the NiO (10 wt%)/SrTiO_3_ sample is significantly lower than that of pure SrTiO_3_. Since a lower PL intensity typically indicates a higher charge separation efficiency [56], this suggests that the recombination of photogenerated electrons and holes is effectively suppressed in the NiO (10 wt%)/SrTiO_3_ sample. This is probably attributed to the formation of an S-scheme charge transfer pathway at the NiO/SrTiO_3_ junction [32]. Consequently, more charge carriers become available to participate in photocatalytic reactions.

**Figure 8:**
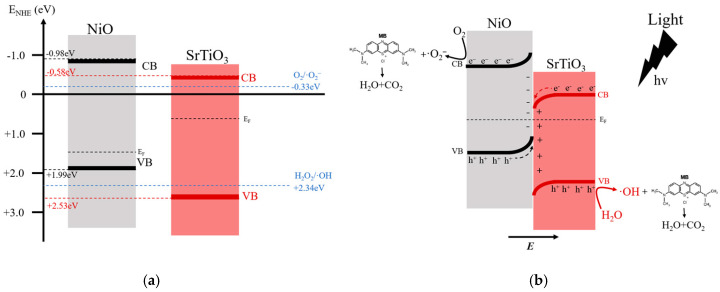
(**a**) Schematic diagram of the band structure of NiO (10%)/STO. (**b**) Proposed photocatalytic mechanism for MB degradation over NiO (10%)/STO.

Based on the above results, a possible photocatalytic mechanism is proposed. Figure 8 shows the schematic illustration of energy band positions before and after junction formation [51,57]. When NiO and SrTiO_3_ contact, a p-n heterojunction is established. Electrons (e^−^) migrate from SrTiO_3_ to NiO, while holes (h^+^) move in the opposite direction. This bidirectional charge transfer generates a built-in electric field at the interface [36]. Due to the mismatch of Fermi levels (E_F_), band bending occurs at the interface until E_F_ reaches equilibrium, resulting in an upward bending of the bands in NiO and a downward bending in SrTiO_3_. Under light irradiation, electrons (e^−^) are excited from the valence band (VB) to the conduction band (CB), generating holes (h^+^) in the VB [57]. The formation of the built-in electric field facilitates the recombination of low-energy photogenerated carriers [32]. This means that the electrons (e^−^) in the CB of SrTiO_3_ recombine with the holes (h^+^) in the VB of NiO, leaving behind electrons in the CB of NiO (−0.98 eV) and holes in the VB of SrTiO_3_ (+2.53 eV). Both types of carriers possess strong redox potentials, making them suitable for photocatalytic reactions. The e^−^ in the CB of NiO can react with dissolved O_2_ molecules in the MB solution, reducing them to superoxide radicals (•O_2_^−^), while the h^+^ in the VB of SrTiO_3_ can oxidize H_2_O to generate hydroxyl radicals (•OH) [58]. These reactive oxygen species subsequently attack the complex structure of MB, breaking it down into CO_2_ and water. The standard redox potentials for the O_2_/•O_2_^−^ pair and the H_2_O/•OH pair are −0.33 eV and +2.34 eV (vs. NHE), respectively. Therefore, the conduction band electrons must possess a potential more negative than −0.33 eV to effectively reduce oxygen, and the valence band holes must have a potential more positive than +2.34 eV to oxidize water [59]. In the NiO (10 wt%)/SrTiO_3_ heterojunction system, the potential of the conduction band minimum of NiO and the valence band maximum of SrTiO_3_ meet these requirements, enabling the generation of both superoxide ions and hydroxyl radicals. This strongly supports the operation of an S-scheme charge transfer pathway, where charge separation is enhanced while high redox capability is maintained [60,61].

## 4. Conclusions

This study proposed a NiO/SrTiO_3_ p-n heterojunction photocatalyst to photodegrade the refractory organic pollutant methylene blue (MB) from water bodies. A series of NiO/SrTiO_3_ samples with different NiO loadings were successfully prepared via a hydrothermal method combined with thermal treatment. The heterojunction structure is formed between NiO and SrTiO_3_, which extends the light-response range into the visible region and significantly suppresses the recombination of photogenerated charge carriers. The optimal sample, NiO (10%)/SrTiO_3_, achieved complete degradation of MB within 9 min, with an apparent rate constant of 0.3813 min^−1^—34.6 times that of pure SrTiO_3_—while demonstrating good cycling stability. Radical trapping experiments confirmed that •OH and •O_2_^−^ are the primary active species in the degradation process. Based on band structure analysis and photoluminescence spectroscopy, an S-scheme charge-transfer mechanism is proposed, elucidating the critical role of the built-in electric field at the heterojunction interface in promoting carrier separation while maintaining high redox capability. Overall, the photocatalytic enhancement originates from the synergistic interplay between preserved crystal structure, interfacial electronic interaction, and heterojunction-driven charge separation. This research provides a new pathway for designing efficient and stable SrTiO_3_-based heterojunction photocatalysts, showing promising application potential for the treatment of organic wastewater.

## Figures and Tables

**Table 1:** Comparison of the photocatalytic performance of SrTiO_3_-based photocatalysts for organic pollutant degradation.

Material	Light Source	Time (min)	Pollutant	Degradation Rate (%)	Reference
NiO/SrTiO_3_	Xenon lamp	9	Methylene blue (10 ppm)	100%	This work
ZnIn_2_S_4_/SrTiO_3_	Xenon lamp	32	Methyl Orange (10ppm)	97.4%	[48]
TiO_2_/SrTiO_3_	Visible irradiation	60	Tetracycline hydrochloride (30 ppm)	98%	[49]
Cu_2_O/SrTiO_3_	Xenon lamp	60	Methylene blue (10 ppm)	90.4%	[50]
PPy/SrTiO_3_	Mercury lamp	90	Rhodamine B (10 ppm)	97.6%	[51]
Bi_2_WO_6_/SrTiO_3_	Xenon lamp	100	Rhodamine B (8 ppm)	71.1%	[52]
WO_3_/SrTiO_3_	Xenon lamp	120	Nitenpyram (40 ppm)	100%	[53]
SrCO_3_/SrTiO_3_	Xenon lamp	120	Tetracycline hydrochloride (20 ppm)	90.5%	[54]

## Data Availability

The raw data supporting the conclusions of this article will be made available by the authors on request.

## Supplementary Materials

The following supporting information can be downloaded at: https://www.mdpi.com/article/10.3390/ma19050845/s1, Figure S1. Statistical analysis of particle size: (a) SrTiO_3_; (b) NiO; Figure S2. The TEM image of SrTiO_3_ sample; Figure S3. Photocatalytic degradation performance of NiO/SrTiO_3_ samples: (a) SrTiO_3_; (b) NiO; (c) NiO (1%)/SrTiO_3_; (d) NiO (5%)/SrTiO_3_; (e) NiO (10%)/SrTiO_3_; (f) NiO (15%)/SrTiO_3_.
