# Supplementary material for: Constructing an S-Scheme NiO/SrTiO3 Heterojunction for Highly Enhanced Visible-Light Photocatalytic Removal of Methylene Blue
Source: Materials (Basel). 2026 Feb 25;19(5):845. doi: 10.3390/ma19050845 (PMC12986170; doi:10.3390/ma19050845)
Supplement: Supplementary file 1 [file materials-19-00845-s001.zip › materials-4154520-supplementary.pdf]

Supporting information for

Constructing an S-Scheme $NiO/SrTiO_3$ Heterojunction for Highly Enhanced Visible-Light Photocatalytic Removal of Methylene Blue

Hongfei Wu [1], Yanlong Gao [1], Senwei Wu [1], Xiujian Zhao [2], Yi Xie [2] and Shouqin Tian [1,*]

[1] State Key Laboratory of Advanced Glass Materials, Wuhan University of Technology, Wuhan 430070, China; 13753131311@163.com (H.W.); 15164661794@163.com (Y.G.); senwei_wu@whut.edu.cn (S.W.)
[2] State Key Laboratory of Silicate Materials for Architectures, Wuhan University of Technology, Wuhan 430070, China; opluse@whut.edu.cn (X.Z.); xiey@whut.edu.cn (Y.X.)
* Correspondence: tiansq@whut.edu.cn

Trapping Experiment

In the radical trapping experiments, isopropanol (IPA) and benzoquinone (BQ) were employed as scavengers for hydroxyl radicals (•OH) and superoxide radicals ($•O_2^-$), respectively. Specifically, 1.0 mL of IPA (corresponding to a concentration of approximately 0.26 mol L^{-1}) and 4 μL of BQ (final concentration of 1 mmol L^{-1}) were added into the reaction system prior to light irradiation. 50 mg of the photocatalyst was dispersed in a quartz glass reactor containing 50 mL of an aqueous methylene blue (MB) solution (initial concentration: 10 mg L^{-1}, pH = 7). A 500 W xenon lamp was used as the simulated solar light source. The mixture was first stirred in the dark for 30 min to establish an adsorption-desorption equilibrium. During the illumination period, approximately 3 mL of the suspension was sampled at 3-min intervals and immediately filtered through a syringe filter with a 0.22 μm membrane to remove the catalyst particles. The degradation of methylene blue was evaluated by monitoring the decrease of its characteristic absorption peak at 667 nm using a UV-Vis spectrophotometer.

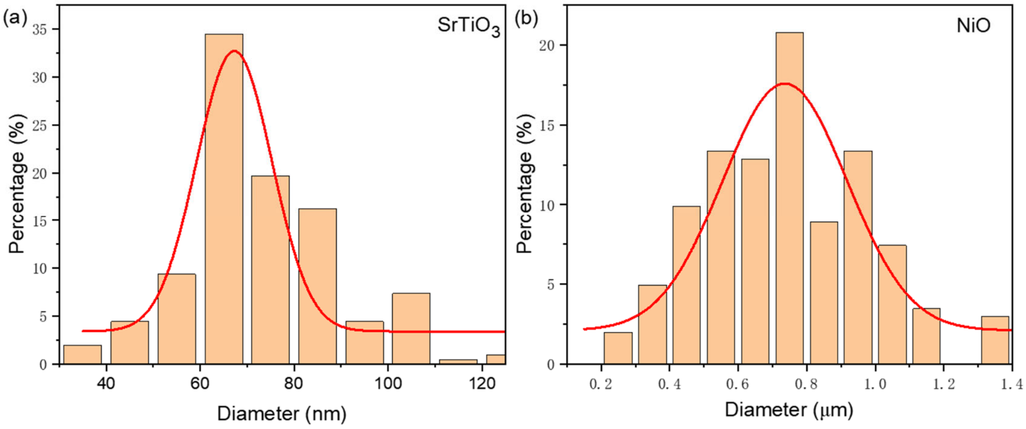

Figure S1 Statistical analysis of particle size: (a)$SrTiO_3$; (b)NiO

To further quantify the particle size of the samples, systematic particle size statistical analysis (statistical sample size > 200) was performed based on SEM images for $SrTiO_3$ and NiO sample. The particle size distribution histograms (Figure S1) indicate that the $SrTiO_3$ particles possess an average size of approximately 60 nm, exhibiting a relatively uniform nanoscale distribution. In contrast, the NiO component predominantly forms plate-like structures with characteristic dimensions of approximately 700 nm. This hierarchical size distribution is beneficial for photocatalytic performance. The nanoscale $SrTiO_3$ provides a high specific surface area and abundant active sites, while the larger NiO plates facilitate effective interfacial contact and charge transport.

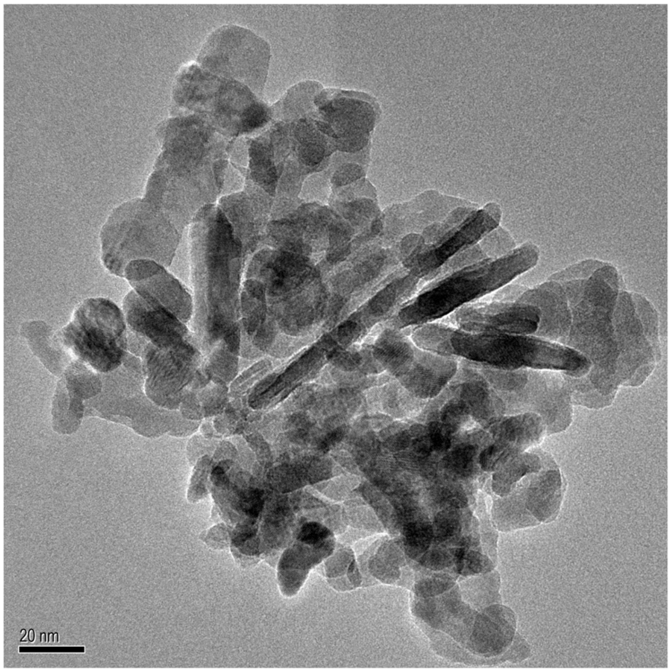

Figure S2 The TEM image of $SrTiO_3$ sample.

The TEM image of the $SrTiO_3$ sample (Figure S2) further confirms the short rod-like morphology and dense aggregation of the $SrTiO_3$ particles. The relatively small particle size implies the presence of abundant exposed surface active sites.

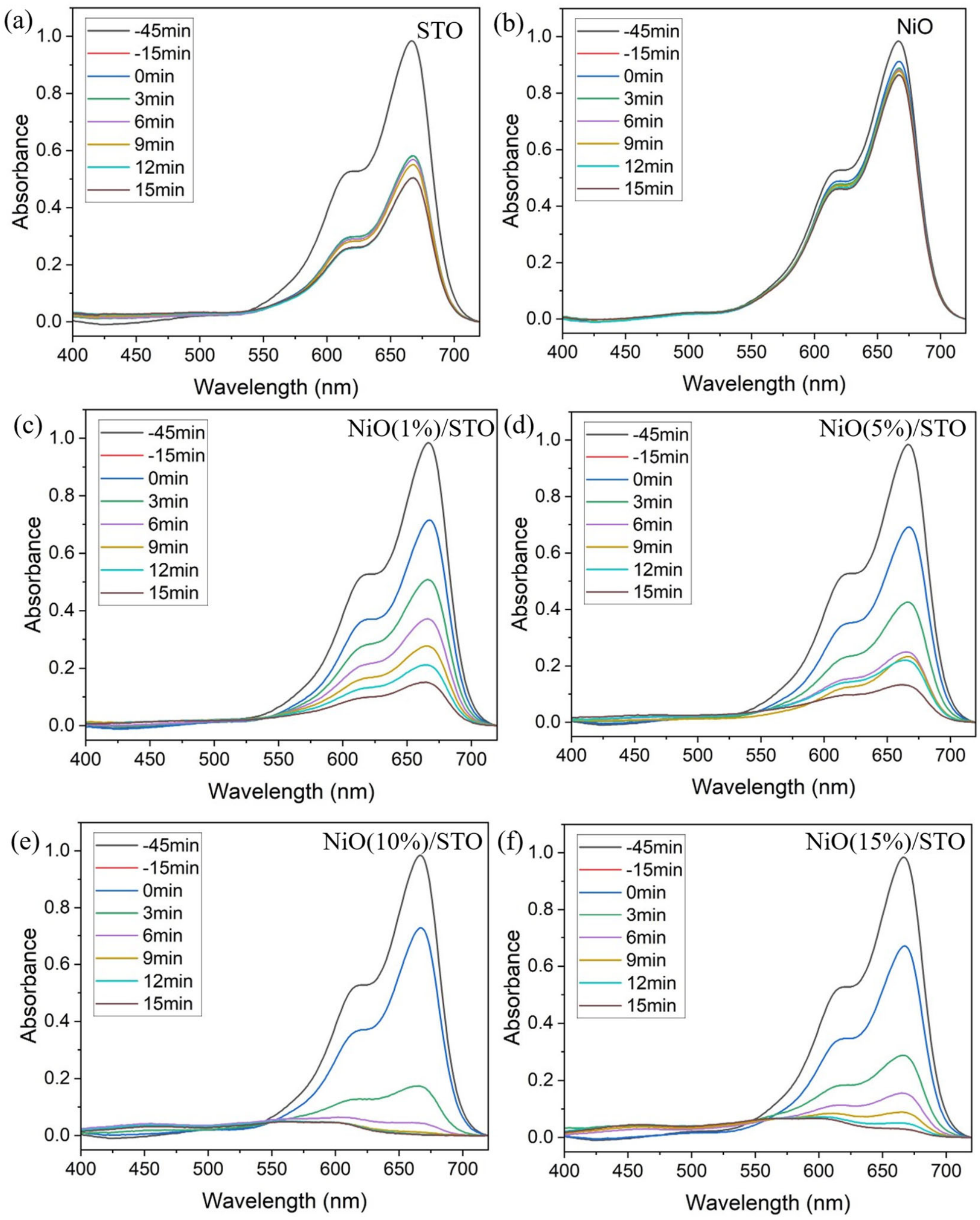

Figure S3 Photocatalytic degradation performance of NiO/$SrTiO_3$ samples: (a)$SrTiO_3$; (b)NiO; (c) NiO(1%)/$SrTiO_3$; (d) NiO(5%)/$SrTiO_3$; (e) NiO(10%)/$SrTiO_3$; (f) NiO(15%)/$SrTiO_3$.
